# Niche partitioning and individual specialisation in resources and space use of sympatric fur seals at their range margin

**DOI:** 10.1007/s00442-024-05537-8

**Published:** 2024-04-03

**Authors:** Marcus Salton, Vincent Raoult, Ian Jonsen, Robert Harcourt

**Affiliations:** 1 School of Natural Sciences, Macquarie University, North Ryde, NSW 2109 Australia; 2 School of Environmental and Life Sciences, University of Newcastle, Ourimbah, 2258 Australia; 3 Present Address: Australian Antarctic Division, Department of Climate Change, Energy, the Environment and Water, Kingston, TAS 7050 Australia

**Keywords:** Competition, Stable isotopes, Foraging ecology, Marine predator, Recovery

## Abstract

Ecological theory predicts niche partitioning between high-level predators living in sympatry as a mechanism to minimise the selective pressure of competition. Accordingly, male Australian fur seals *Arctocephalus pusillus doriferus* and New Zealand fur seals *A. forsteri* that live in sympatry should exhibit partitioning in their broad niches (in habitat and trophic dimensions) in order to coexist. However, at the northern end of their distributions in Australia, both are recolonising their historic range after a long absence due to over-exploitation, and their small population sizes suggest competition should be weak and may allow overlap in niche space. We found some niche overlap, yet clear partitioning in diet trophic level (δ^15^N values from vibrissae), spatial niche space (horizontal and vertical telemetry data) and circadian activity patterns (timing of dives) between males of each species, suggesting competition may remain an active driver of niche partitioning amongst individuals even in small, peripheral populations. Consistent with individual specialisation theory, broad niches of populations were associated with high levels of individual specialisation for both species, despite putative low competition. Specialists in isotopic space were not necessarily specialists in spatial niche space, further emphasising their diverse individual strategies for niche partitioning. Males of each species displayed distinct foraging modes, with Australian fur seals primarily benthic and New Zealand fur seals primarily epipelagic, though unexpectedly high individual specialisation for New Zealand fur seals might suggest marginal populations provide exceptions to the pattern generally observed amongst other fur seals.

**Supplementary Information:**

The online version contains supplementary material available at 10.1007/s00442-024-05537-8.

## Introduction

Understanding the factors that limit species’ distributions is a key theme in ecology. An important factor that limits the distribution of many plants and animals is interrelations amongst species which determine food supply, threat of predation, disease and competition (Krebs 2001). In the case of competition, two species living in a community can compete for resources to a point where one species compromises the fitness of another, but can coexist by partitioning resources or risk competitive exclusion (MacArthur and Levins 1967; Pacala and Roughgarden 1982; Luiselli 2006). Interspecific competition is ubiquitous in plants and animals, though particularly prevalent at higher trophic levels and/or amongst larger animals where available resources may be more limited (Connell 1983; Schoener 1983). Many populations of large carnivores are currently recovering and expanding their range due to persistent conservation efforts (Wabakken et al. 2001; Chapron et al. 2014; Gompper et al. 2015; Martinez Cano et al. 2016). During such recoveries, the interrelations with species in the existing community and with other recovering carnivores are often unknown, but can involve interspecies competition with detrimental impacts to some species, including human conflict (Gompper 2002; Thornton et al. 2004; Kilgo et al. 2010; Reddy et al. 2019; Engebretsen et al. 2021; Franchini et al. 2021). Therefore, determining factors that mitigate competition and mechanisms for coexistence remain important in ecology and will support conservation management.

Niche theory suggests it is possible for competing species to coexist if they occupy different niches (Hardin 1960; MacArthur and Levins 1967). Within a species, similar individuals manage to coexist by partitioning resources, with individuals that have contrasting morphology, physiological capacity, energy requirements or social status typically adopting different strategies to exploit available resources (Svanbäck and Bolnick 2007). Individuals can also use a subset of the population’s resources for reasons unrelated to sex, age and morphological variation, i.e. inter-individual variation (Bolnick et al. 2003; Araújo et al. 2011), with more specialised individuals using a smaller subset and more generalised individuals using a larger subset of the population resources. The level of inter-individual variation can be positively related to population density—a proxy for intraspecies competition (Svanbäck and Persson 2004; Svanbäck and Bolnick 2005, 2007; Araújo et al. 2008; Tinker et al. 2012; Newsome et al. 2015). At the edge of a species’ geographic range, population size is small and thereby intraspecies competition tends to be low, reducing pressures associated with population density, but here interspecies competition with a novel community can be an important factor setting range limits (Hersteinsson and Macdonald 1992; Case and Taper 2000; Case et al. 2005; Pigot and Tobias 2013).

By progressing the study of how species coexist, particularly at expanding margins of their range, we can better assess and predict the interrelations between species as they recover and move into new communities. There are now well-established methods for quantifying ecological niche size and partitioning, including variance and ellipse-based metrics, and spatial, resource and temporal dimensions (Pielou 1972; Petraitis 1979; Bearhop et al. 2004; Peres-Neto et al. 2006; Jackson et al. 2011; Swanson et al. 2015; Frey et al. 2017) have been used to demonstrate that individuals can coexist by partitioning parts of their niche space, resources and time (Luiselli 2006; Navarro et al. 2013; Dehnhard et al. 2020). These niche dimensions have often been assessed in isolation, but with the proliferation of stable isotope analyses and telemetry more studies are demonstrating the importance of a multifaceted approach to understanding niche partition (Kleynhans et al. 2011; Matich and Heithaus 2014; Baylis et al. 2015; Giménez et al. 2018; Riverón et al. 2021; Schwarz et al. 2021). There have also been advances in measuring intra- and interspecific variability in resource and space use (Bolnick et al. 2002; Araújo et al. 2007; Zaccarelli et al. 2013; Carneiro et al. 2017; Bonnet-Lebrun et al. 2018) that require serial sampling individuals to determine individual specialisation (Newsome et al. 2010; Eerkens et al. 2016). Animals can be monitored over long periods of time by combining telemetry with sampling tissues that accumulate isotopes, with both approaches capable of quantifying individual specialisation (Bearhop et al. 2006; Newsome et al. 2009; Elorriaga-Verplancken et al. 2013; Kernaléguen et al. 2016; Bonnet-Lebrun et al. 2018). Commonly analysed isotopes include nitrogen, as an indicator of trophic position of prey, and carbon, as an indicator of geographic origin of prey (Kelly 2000; McCutchan et al. 2003). In marine systems, carbon isotopes can reflect nearshore vs. offshore foraging and prey originating from benthic vs. epipelagic environments (Michener and Kaufman 2007; Newsome et al. 2010). Therefore, the tools are now available to provide detailed assessments of how previously exploited large predators coexist as they recover and expand their range.

Otariids, fur seals and sea lions, were universally overharvested for their fur from the eighteenth to twentieth century, with extinction of many populations and dramatic range reductions (Bonner 1989; Gerber and Hilborn 2001). With persistent conservation efforts, many species have been recovering in recent decades and reoccupying parts of their historic ranges (Wickens and York 1997; Gerber and Hilborn 2001; Kirkman et al. 2013; Crespo 2021; Salton et al. 2021). There are many incidences of two otariid species living in sympatry during such recoveries (Majluf and Trillmich 1981; Lyons et al. 2000; Wege et al. 2016; Elorriaga-Verplancken et al. 2021), and whilst this seems to be possible by niche partitioning (Robinson 2002; Franco-Trecu et al. 2012; Páez-Rosas et al. 2012; Jeglinski et al. 2013; Pablo‐Rodríguez et al. 2016; Hoskins et al. 2017) different levels of individual specialisations in diet and foraging amongst species may also play a role (Franco-Trecu 2014; Kernaléguen et al. 2015a, 2015b; Riverón et al. 2021). During population recovery, some sympatric species have displayed disparate population growth rates and range expansion, which could be attributed to interrelations between the similar species (Wickens and York 1997; Villegas-Amtmann et al. 2013; Franco-Trecu 2014; Elorriaga-Verplancken et al. 2021).

Here, we investigate how two otariids, the Australian fur seal, *Arctocephalus pusillus doriferus*, and the New Zealand fur seal, *A. forsteri* (also known as long-nosed fur seal, Shaughnessy and Goldsworthy 2015), coexist in sympatry at an expanding margin of both species’ range. These species have recently re-established seasonal occupation of their north-eastern range margins (Warneke 1975; Irvine et al. 1997; Shaughnessy et al. 2001; Burleigh et al. 2008; Salton et al. 2021) following broader population recovery and range expansion (Arnould et al. 2003; Shaughnessy et al. 2015; McIntosh et al. 2018). Their populations at the margins remain small and predominantly consists of juveniles and sub-adult males (Burleigh et al. 2008), though both breed on Montague Island (36° 14′ S, 150° 13′ E), in small numbers (McIntosh et al. 2018). The two species are typically considered ‘generalists’ due to their broad diets (Page et al. 2005a; Kliska et al. 2022), but in some areas, Australian fur seals do exhibit individual specialisations in diet and foraging (Kernaléguen et al. 2012, 2016; Knox et al. 2018). The two species have apparently distinct foraging modes, with Australian fur seals primarily foraging during benthic dives over the continental shelf (Knox et al. 2017; Salton et al. 2019) and New Zealand fur seals foraging during pelagic dives on and off the continental shelf (Page et al. 2005b, 2006; Salton et al. 2021). From two independent studies of diet and foraging behaviour of males in this part of their range (Hardy et al. 2017; Salton et al. 2021), the two species are known to partition parts of their diets and foraging behaviour, though the discrete analyses and absence of any measurement of the extent of overlap in these niche dimensions left uncertainty over what are the actual mechanisms for coexistence. Given the small population sizes of both species, we expect intraspecies competition to be low and, accordingly, relaxation of niche partitioning and greater overlap between species or niche partitioning to be re-enforced at range margins either by interspecies interactions or because individuals are not plastic enough in foraging behaviour to relax constraints imposed at the range core when inhabiting the range margin.

To understand the mechanisms for coexistence in a situation with purported low intraspecies competition, we aim to (1) estimate niche sizes, in isotopic and spatial dimensions, and the degree of partitioning between species at a population level, and (2) the degree of individual specialisation at the intra-population level and how it relates to their population niche size. Then, (3) we assess the relationship between-individual specialisation in isotopic space and individual specialisation in spatial dimensions, and the importance of intrinsic differences in body size.

## Methods

### Ethics statement

All research protocols were conducted under Office of Environment and Heritage Animal Ethics Committee Approval (100322/03) and Macquarie University Ethics Committee Approval 2011/054. Capture and handling methods are detailed in Salton et al. (2021), and included sedation by intra-muscular injection of zoletil using a pneumatic dart-gun, then restraint using a catch net with the animal maintained under sedation with a mix of oxygen and isoflurane delivered via a potable vaporiser. Whilst sedated, standard body length was measured using standard methods (±1 cm, Kirkwood et al. 2006), and the telemetry device (see below) was glued to the dorsal midline of each seal with a quick-setting epoxy (Araldite® K-268, Huntsman Advanced Materials; Quick Set Epoxy Resin 850-940, RS components, Australia). Devices remained on the seals until they fell off, once their fur weakened towards the annual moult. Access to the study site at Jervis Bay was under the guidance and support of the Australian Navy, New South Wales National Parks and Wildlife Service, Jervis Bay Marine Park and the Beecroft Ranger Station. Access to the study site at Montague Island was under the guidance and support of New South Wales National Parks and Wildlife Service.

### Study species, study site and data collection

**Fig. 1:**
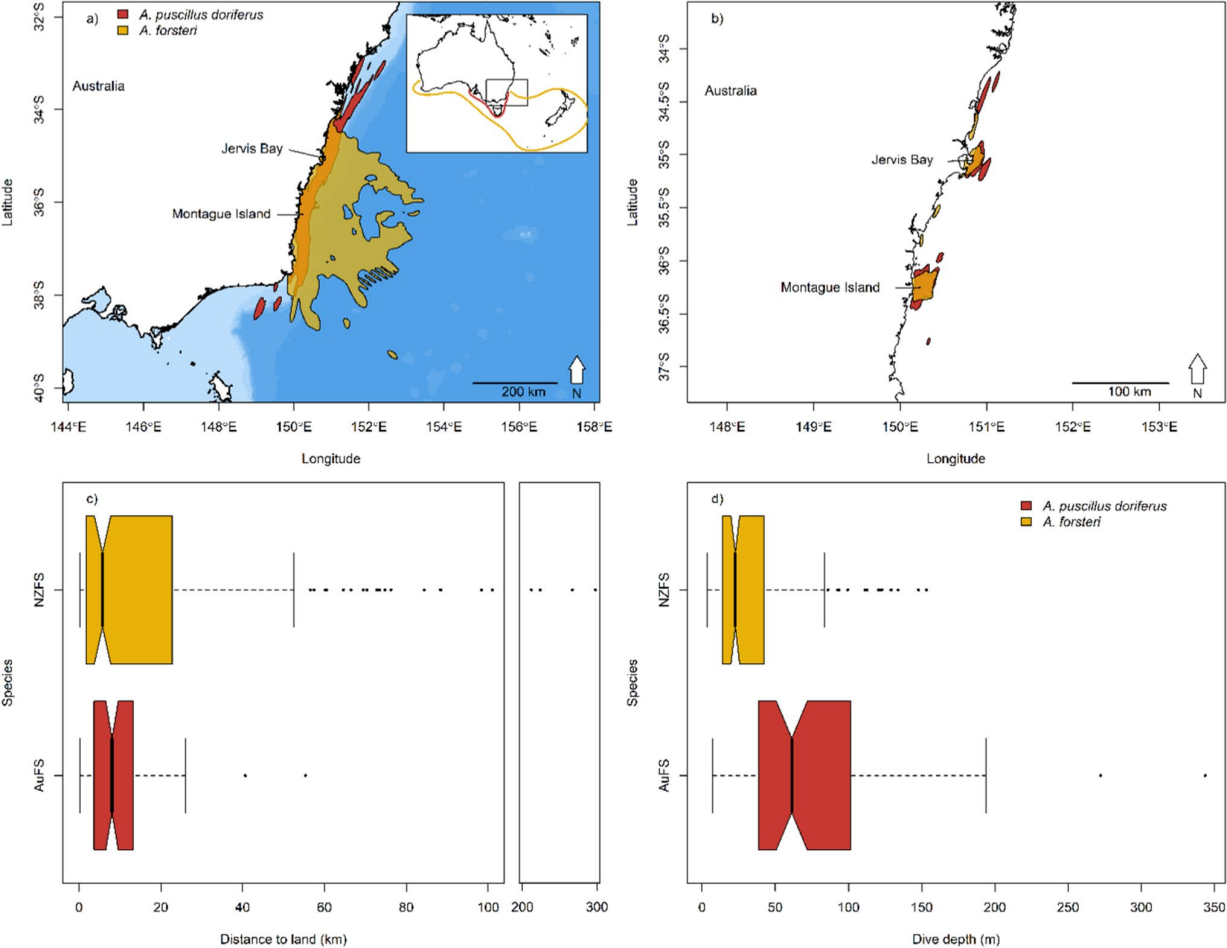
Utilisation distributions **a** 95% **b** 50% and box–whisker plots of spatial niche parameters for male Australian fur seals (*A. pusillus doriferus*, AuFS, *red*; *N* = 10) and New Zealand fur seals (*A. forsteri*, NZFS, *yellow*; *N* = 35 and 39 for dive and location data, respectively) from Jervis Bay and Montague Island (sites combined). Continental shelf (<500 m depth) is light blue. *Inset map* in panel **a** shows approximate range of each species. In panels **c** and **d**, *boxes* represent 1st and 3rd quartiles and median as a *thick line*, and whiskers are ×1.5 inter quartile range. Panel **c** is cropped between 100 and 200 km for clarity (16 points for NZFS not visible). Notches in the *boxes* indicate 95% confidence interval around the median and overlap in notches between groups suggests the medians are not significantly different

The data were collected during the male’s inter-breeding period between 25-May and 22-Aug in 2011 to 2014, inclusive, when they are free of immediate reproductive constraints and, therefore, have no requirement to attend a specific terrestrial site and so can range widely. The breeding period for Australian fur seals is between late October and late December and for New Zealand fur seals between early November and early January (Crawley and Wilson 1976; Warneke and Shaughnessy 1985). Males move away from areas used during their inter-breeding period towards breeding colonies at the approach of breeding seasons, and it is assumed the reverse occurs at the end of breeding, consistent with the seasonal pattern of attendance at these inter-breeding areas (Shaughnessy et al. 2001; Burleigh et al. 2008) and resighted seals marked with flipper tags at colonies (Warneke 1975). Male fur seals were captured at two study sites, Jervis Bay (35° 3′ S, 150° 50′ E) and Montague Island (36° 14′ S, 150° 13′ E) on the southeast coast of Australia (Fig. 1). This coastline has a narrow continental shelf (17–72 km width) with the shelf break between 130 and 170 m (Geoscience Australia, data.gov.au, 2017-06-24). The populations of both fur seal species have recently been growing in this north-eastern region of both species’ range after near extirpation from over harvesting, and at the time of this study, the populations remained small (<150 seals and <20 pups, compared to >10,000 seals and >1500 pups at large colonies; Warneke 1975; Burleigh et al. 2008; Kirkwood et al. 2010; McIntosh et al. 2018).

Movements of males were recorded with Mk10-AF Fastloc-GPS devices (Wildlife Computers; 105 × 60 × 20 mm, 240 g) at Jervis Bay and CTD-SRDL-9000 (Conductivity-Temperature-Depth Satellite Relay Data Logger, Sea Mammal Research Unit, St Andrews, UK; 120 × 72 × 60 mm, 545 g) at Montague Island. Both devices collected Argos satellite-derived locations (collected at irregular time intervals, with a median fix rate of 1 fix per 1.1 h), and Mk10 devices also recorded GPS locations (collected at 2 min intervals, with a median fix rate of 1 fix per 1.5 h), both of which were transmitted via the Argos satellite network (Collecte Localisation Satellites, Saint-Agne, France). Dive data were collected with both devices (but not Mk10-AF in 2011), with depth (±0.5 m) sampled every 5 s when the device was wet. Single dives were defined by a minimum depth of 5 m and minimum duration of 10 s, and if these criteria were exceeded, the maximum depth of the dive was recorded.

To account for potential inter-annual variability in resource use (Rodríguez-Malagón et al. 2021), we sampled individual vibrissae from both species across each year of the study. The longest whisker was sampled (plucked) from each seal whilst a tracking device was being attached. One whisker was sampled from a dead seal incidentally in 21 November 2012. In the laboratory, vibrissae were hand-washed in 100% ethanol and cleaned in an ultrasonic bath of distilled water for 5 min. Vibrissae were then dried, measured and cut into 3 mm-long consecutive sections starting from the proximal (facial) end, following Cherel et al. (2009). The first 10 sections were sampled from all individuals. Vibrissae growth rate estimates for Australian fur seal males are 0.17 ± 0.04 mm d^−1^ (Kernaléguen et al. 2015b), and whilst they are not known for male New Zealand fur seals, we assume it is similar based on growth rate estimates of other male fur seals: *Arctocephalus australis* 0.13 mm d^−1^, *Arctocephalus gazelle* 0.14 ± 0.02 mm d^−1^, and *Arctocephalus tropicalis* 0.14 ± 0.04 mm d^−1^ (Kernaléguen et al. 2012; Vales et al. 2015). Hence, a 3 mm section corresponded to approximately 18 days (Kernaléguen et al. 2015b). The δ^13^C and δ^15^N values of each whisker section were determined by a PDZ Europa ANCA-GSL elemental analyser interfaced to a PDZ Europa 20–20 isotope ratio mass spectrometer (Sercon, Cheshire, UK) at the University of California Davis (UC-Davis) Stable Isotope Facility. Results are presented in the conventional δ notation relative to Vienna PeeDee Belemnite marine fossil limestone and atmospheric N2 for δ^13^C and δ^15^N, respectively. Replicate measurements of internal laboratory standards indicate measurement errors of <0.19%_0_ and <0.26%_0_ for δ^13^C and δ^15^N values, respectively.

### Data processing

All data processing, analysis and figure development were conducted in R v4.1.1 (R Core Team 2020).

Locations were subjected to standard quality-control checks, including removal of erroneous and duplicated locations, removal of locations after a tag fell off a seal, and reclassification of Argos Z-class locations to B-class (*n* = 86/56,978 locations). A continuous-time correlated random walk state-space model (Jonsen et al. 2020) was then fitted to the quality-controlled locations using the *‘fit_ssm’* function in the ‘*foieGras*’ R package (Jonsen and Patterson 2020). This approach accounted for observation errors in the Argos location data, and provided location estimates with standard errors at regular 3 h time intervals along each individual’s track (Jonsen et al. 2013). Foraging ‘distance to land’ was used as an index of horizontal movement behaviour. To calculate this index, SSM-estimated locations were projected using Albers equal-area based on the extent of the seal’s movements, determined using https://projectionwizard.org/, then distance to the Australian coastline (GEODATA Coast 100 K 2004, Geosciences Australia) was calculated using the ‘*gDistance*’ function in the ‘rgeos’ R package (Bivand and Rundel 2021). Locations within 100 m of land were assumed to be indicative of the seal being on land or not foraging and removed.

To best represent the foraging behaviour of animals at the expanding range margins, we analysed only the 10 most recent whisker sections to represent an individual’s isotopic niche and the first 10 weeks of tracking data to represent their spatial niche. This avoided details of their seasonal migrations that may influence the stable isotope values preceding the period at the range margin (Online Resource, Fig. S1; Kernaléguen et al. 2015b; Salton et al. 2021). Based on the whisker growth rate estimates (presented above), the isotope data corresponded to diet approximately 180 days prior to sampling (i.e. approximately the first 6 months of the year). Each whisker section represented a unique sample of δ^13^C and δ^15^N values per individual. In terms of their movements at sea, unlike females, these male fur seals are liberated from the reproductive constraint of returning to a central place to feed pups, and so the vast majority of their movements at sea are likely to be driven by their own foraging needs, and these needs are motivated by selective pressure to attain a large, competitive body size to enable access to females. Thus, for movement data, distance to land and maximum dive depth were averaged per week for each individual, and these weekly averaged values represented individual samples of movement behaviour.

### Niche partitioning and individual specialisation

Species differences in the two isotope variables (δ^13^C and δ^15^N) and two spatial variables (distance to land and dive depth) were tested using linear mixed models. For each of the four variables, a linear mixed model was fitted with species a fixed categorical effect and sample nested in individual identity as a random effect, using the ‘*lme*’ function in the ‘nlme’ R package (Pinheiro et al. 2021). All models included a temporal autocorrelation (corAR1 of form ~1|ID) to account for serial sampling of individuals. When there were model convergence issues (i.e. δ^15^N), these were corrected by removing the nested sample component of the random effect. Akaike Information Criterion (AIC) and analysis of variance tests were used to compare the model with fixed effects to the null model, with *P* < 0.05 indicative of a significant difference from the null model, following the protocol outlined by Zuur et al. (2009). We tested the interactions between species and year and species and deployment site to account for these possible spatial and temporal variations in the isotopes (Rodríguez-Malagón et al. 2021). A weak species:year interaction effect for carbon isotope (conditional *R*^2^ = 0.14, *P* = 0.014) was apparent, but no year effect for each species. Given their absence or that they were weak, we did not include them in subsequent models.

Distance to land and dive depth were log-transformed to account for these indexes being highly positively skewed, and the model estimates are presented back-transformed with their confidence interval (alternatively, isotope estimates are presented with their modelled standard error).

The 95% and 50% spatial utilisation distribution (UD) probabilities were calculated for the inter-breeding period. Smoothing parameters for the UD were calculated using the plug-in bandwidth selector function ‘*Hpi*’ and associated ‘*kde*’ function in the ‘*ks*’ R package (Duong 2021), and the Australian coastline was used as a habitat grid to ensure realistic UD probabilities over water. UDs were calculated for each individual and then standardised to produce a population level 95% and 50% UD for AuFS and NZFS. Percentage UD overlap was calculated using the equation [(area_*ab*_/UD_*a*_) × (area_*ab*_/UD_*b*_)]^0.5^, where area_*ab*_ is the area of overlap in the home ranges of species *a* and *b*, and UD_*a*_ and UD_*b*_ refer to the UD of species *a* and *b*, respectively (Atwood et al. 2003; Hoskins et al. 2017).

To test for partitioning in the circadian pattern of dive behaviour, we assessed whether dive frequency and dive depth differed with three diel periods: day, twilight and night. Solar position was calculated using solar azimuth and elevation based on location, local date and time (Australian eastern standard time: UTC + 10 h), using the ‘*solarpos*’ function in the ‘*maptools*’ R package (Bivand and Lewin-Koh 2021). From solar position, a categorical variable for diel period was defined with three levels: positive values of solar elevation angle identified ‘day’; values between zero and −12° below the horizon identified nautical ‘twilight’; and values below −12° identified ‘night’. Generalised linear mixed models were fitted to assess whether dive frequency was explained by diel period, for each species separately, using the ‘*lmer*’ function in the ‘*lme4*’ R package (Bates et al. 2015) with a random effect for individual (intercept only, to elevate convergence issues with the models) and a Poisson error distribution with a log link function. Linear mixed models were fitted to assess whether dive depth (log-transformed) was explained by diel period, for each species separately, using the ‘*lmer*’ function in the ‘*lme4*’ R package (Bates et al. 2015) with a random effect for individual (intercept only, to elevate convergence issues with the models). AIC and analysis of variance were again used to compare the model with fixed effects to the null model, with *P* < 0.05 indicative of a significant difference from the null model.

Isotopic and spatial niche size and partitioning between species were estimated using Bayesian ellipse-based metrics calculated in the ‘SIBER’ R package (Jackson et al. 2011). SIBER applies a ‘typical’ individual approach to calculate the core niche of a population, and incorporates uncertainties relating to sampling biases and small sample sizes, including robust comparison amongst datasets of different sample size (Jackson et al. 2011; Syväranta et al. 2013). We used the 40% Bayesian standard ellipse area (SEA_b_) to represent the most reliable population-level niche, with the variance estimated through 10^4^ posteriori draws, and a 95% SEA_b_ to capture individual variation and enable more accurate cross-study comparisons. Repeated sample measurements per individual were not independent, yet the small sample size of individual Australian fur seals produced highly variable niche estimates for that population, albeit with consistent niche size compared to the whole dataset (Sup 1). Independent sampling is a required assumption for use of Bayesian SEA_b_ (Jackson et al. 2011), but incorporating a large number of individuals as in this case was preferable to other methods of assessing isotope niche. SEA_b_ results should nevertheless be interpreted in combination with results from mixed effect models. Overlap of isotopic and spatial niches was calculated per species based on the posterior distributions of the fitted ellipses using the ‘*baysianOverlap*’ function (*n* = 360, draws = 50).

The degree of individual specialisation in male AuFS and NZFS for each of the four niche parameters was measured and compared using Roughgarden’s WIC/TNW index for continuous data (Bolnick et al. 2002). The approach considers the total niche width (TNW), or variance in total niche parameter for all individuals, to be a sum of the within-individual component (WIC) and the between-individual component (BIC). The WIC is the average of individual niche widths, for example, the variance in isotopes within each individual’s whisker, and the BIC is the variance in mean parameter estimates (e.g. isotope values) amongst individuals. The ‘WTcMC’ function in the ‘RInSp’ R package (Zaccarelli et al. 2013) was used to calculate the specialisation index (SI) for each population, weighting each individual equally to account for slight variances in the number of samples per individual. The SI varied between 0 (specialist) and 1 (generalist), and we applied Monte Carlo resampling (using 1000 replicates) to test the null hypothesis that all individuals were sampled equally from a generalist population. Relationships between the SI for the four niche parameters and with individual body length were tested using linear models, separately for each species, with t-statistics used to assess the fitted linear model, with *P* < 0.05 indicative of a significant relationship. A lack of relationship between the SI of each niche parameter and body size ensured the measure of individual specialisation aligned with the definition by Bolnick et al. (2002).

## Results

We analysed 9 vibrissae from male Australian fur seals (AuFS), with 10 3 mm segments from each vibrissae, and 35 vibrissae from male New Zealand fur seals (NZFS), with 8, 9 or 10 3 mm segments from each vibrissae. Location and dive recording data were retrieved from 10 male AuFS and 35 male NZFS, and location data with no dive recording data were retrieved from an additional four male NZFS. Location and dive data were recorded for 15–259 days (mean ± SE 131.9 ± 15.5 days and 101.4 ± 10.7 days per individual, respectively), which was equivalent to 15 ± 1.2 weeks with location and dive data, 635 ± 53 locations (from SSM, at 3 h intervals) and 1151 ± 221 dives per individual. Based on body length of the seals, male AuFS were larger than male NZFS (body length mean ± SE 192 ± 7.9 cm, *N* = 9 individual, vs. 137 ± 5.7, *N* = 39 individuals, respectively; Wilcoxon rank sum test *W* = 339, *P* < 0.001).

### Isotopic and spatial niche

**Fig. 2:**
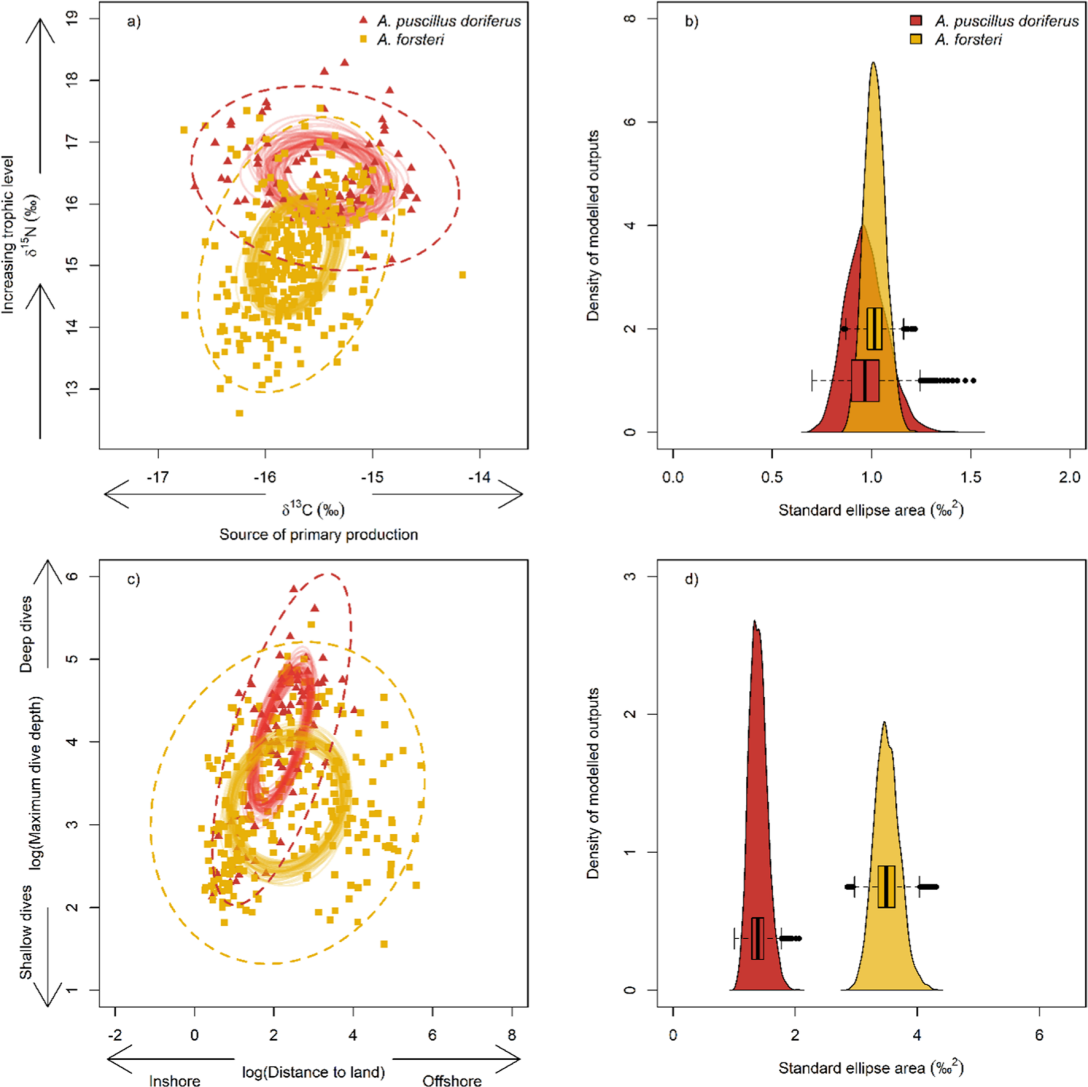
Isotopic and spatial niche bi-plots (*left*) and posterior density plots (*right*) from Bayesian standard ellipse area (SEA_b_; *solid lines* 40%, *dashed line* 95%; density plots are of 40% SEA_b_) of male Australian fur seals (*Arctocephalus pusillus doriferus*, *red*; *N* = 9) and New Zealand fur seals (*A. forsteri*, *yellow*; *N* = 35). In isotope bi-plot, points represent isotope values from the ten most recent whisker samples from each individual. For clarity, a sample of 50 modelled ellipses (40% SEA_b_) per species are shown. Bi-plots represent the size and overlap of the niche space, and density plots compare size (similar niche size have more overlap) and variance amongst 40% SEA_b_ estimates (height-width of density plot)

The two species had broad, segregated isotopic niches of similar size. There were significant differences in δ^15^N and δ^13^C values between male AuFS and NZFS, with AuFS having higher δ^15^N values (AuFS mean 16.4 ± 0.2; NZFS mean 15.2 ± 0.2) and higher but ecologically similar δ^13^C values (AuFS mean −15.4 ± 0.2; NZFS mean 15.2 ± 0.2) (models were significantly different to the null model, δ^15^N Δ*AIC* = 17.65 *χ*^2^ = 19.65 *P* < 0.001; δ^13^C Δ*AIC* = 3.16 *χ*^2^ = 5.16 *P* = 0.023; Table 1). Australian fur seals had a narrower range of δ^15^N values (trophic levels; AuFS 15.91–17.55; NZFS 13.92–16.59) and wider range of δ^13^C (nutritional sources; AuFS −15.98 to −14.74; NZFS −16.21 to −15.25) compared to New Zealand fur seals (Fig. 2; Table 1). Bayesian estimation of the isotopic niche space of the two species shows similar sized isotopic niches, based on the 40% SEA_b_ and 95% SEA_b_, yet trophic niche (40% SEA_b_) overlap was negligible at ~5%, suggesting strong resource partitioning between the two pinniped populations. Based on the 40% SEA_b_, partitioning of their iso-niche space was primarily in δ^15^N values that relate to trophic level (Fig. 2; Table 1).

**Table 1:** Predicted mean estimates for δ^13^C and δ^15^N isotopes (±SE) and the two spatial niche statistics ‘dive depth’ and ‘distance to land’ (with confidence intervals) of male Australian fur seals (*A. pusillus doriferus*) and New Zealand fur seals (*A. forsteri*) from linear mixed effects models

Population-level statistics	*A. pusillus doriferus*	*A. forsteri*
*Isotope niche space*		
δ^15^N (‰)^a^ mean	16.4 ± 0.2	15.2 ± 0.2
Range	(15.39 to 18.69)	(13.48 to 17.09)
δ^13^C (‰)^a^ mean	− 15.4 ± 0.1	− 15.7 ± 0.1
Range	(−16.70 to −13.79)	(−17.57 to −13.98)
Number of seals (*N*) and whisker segments (*n*)	***N* = *9, n* = *90***	***N* = *35, n* = *345***
*Spatial niche space*		
Maximum dive depth (m)^b^	58.1, CI 35.5 to 85.5	25.3, CI 9.0 to 68.1
Number of seals (*N*) and dives (*n*)	***N* = *10, n* = *95***	***N* = *35, n* = *275***
Distance to land (km)^b^	6.3, CI 3.4 to 11.0	6.3, CI 1.4 to 20.6
Number of seals (*N*) and weeks of tracking (*n*)	***N* = *10, n* = *97***	***N* = *39, n* = *332***

Isotopes calculated from 3 mm segments from one vibrissae per individual. Movement statistics calculated from weekly mean statistics per individual. Individual identity was included as a random effect in the models

^a^Mean ± SE and range are calculated at the individual level (i.e. mean of each individual’s average value for its whisker segments or weekly movement data)

^b^Spatial niche parameters were log-transformed, and subsequently their back-transformed estimates of means are accompanied by 95% confidence intervals (CI)

Male AuFS remained close to the coast over the continental shelf, including over the shelf edge, whilst NZFS travelled across the continental shelf (coastal and over the shelf edge) and well off the shelf over deep water. Consequently, male NZFS had a much larger 95% utilisation distribution than AuFS (Table 1), and the percentage overlap or 95% UD shared with the other species was ~80% for AuFS and ~10% for NZFS. However, the 50% UD for both species was predominantly over the continental shelf, of similar size, and showed approximately 50% species overlap (Fig. 1; Table 1). Accordingly, the mean distance that an individual travelled from land per week was highly positively skewed for male AuFS and NZFS, and not significantly different between the two species (distance to land not significantly different to the null model, Δ*AIC* = 1.6 *χ*^2^ = 0.32 *P* = 0.574; Table 1). The two species also shared the vertical dimension of their spatial niche, but on average male AuFS dived deeper than NZFS (dive depth significantly different to the null model, Δ*AIC* = 7.9 *χ*^2^ = 9.89 *P* = 0.002; Fig. 1; Table 1). The movement behaviour of AuFS (i.e. predominantly deep dives over the continental shelf) was consistent with a benthic foraging mode, and the movement behaviour of NZFS (shallow dives over the shelf and deep water) was consistent with epipelagic foraging mode. However, four male NZFS with weekly average maximum depth >100 m also remained close to land (<20 km) during those weeks, suggesting benthic foraging; this was the case for all weeks recorded for one of these four NZFS, suggesting it used a benthic foraging mode during the inter-breeding period.

With horizontal and vertical movements combined, overall NZFS had a much larger spatial niche space (40 and 95% SEA_b_; Table 1). Whilst they had overlapping spatial niche space, AuFS shared more of their spatial niche space with NZFS, but due to more extensive horizontal movements, NZFS had more space segregated from AuFS space. At the same time, whilst the larger spatial niche space for NZFS derived from horizontal movement, partitioning in spatial niche space between species was primarily due to segregation in vertical use of the water column, as Australian fur seals dived deeper and New Zealand fur seals dived shallower (Fig. 2).

The two species also had different circadian patterns in dive frequency, with NZFS diving significantly more at night and AuFS diving similarly between night and day, but significantly less during twilight (Online Resource; Fig. S2, Table S1). Neither species had a diel pattern in dive depth (Online Resource; Fig. S2, Table S1).

#### Individual specialisation

**Fig. 3:**
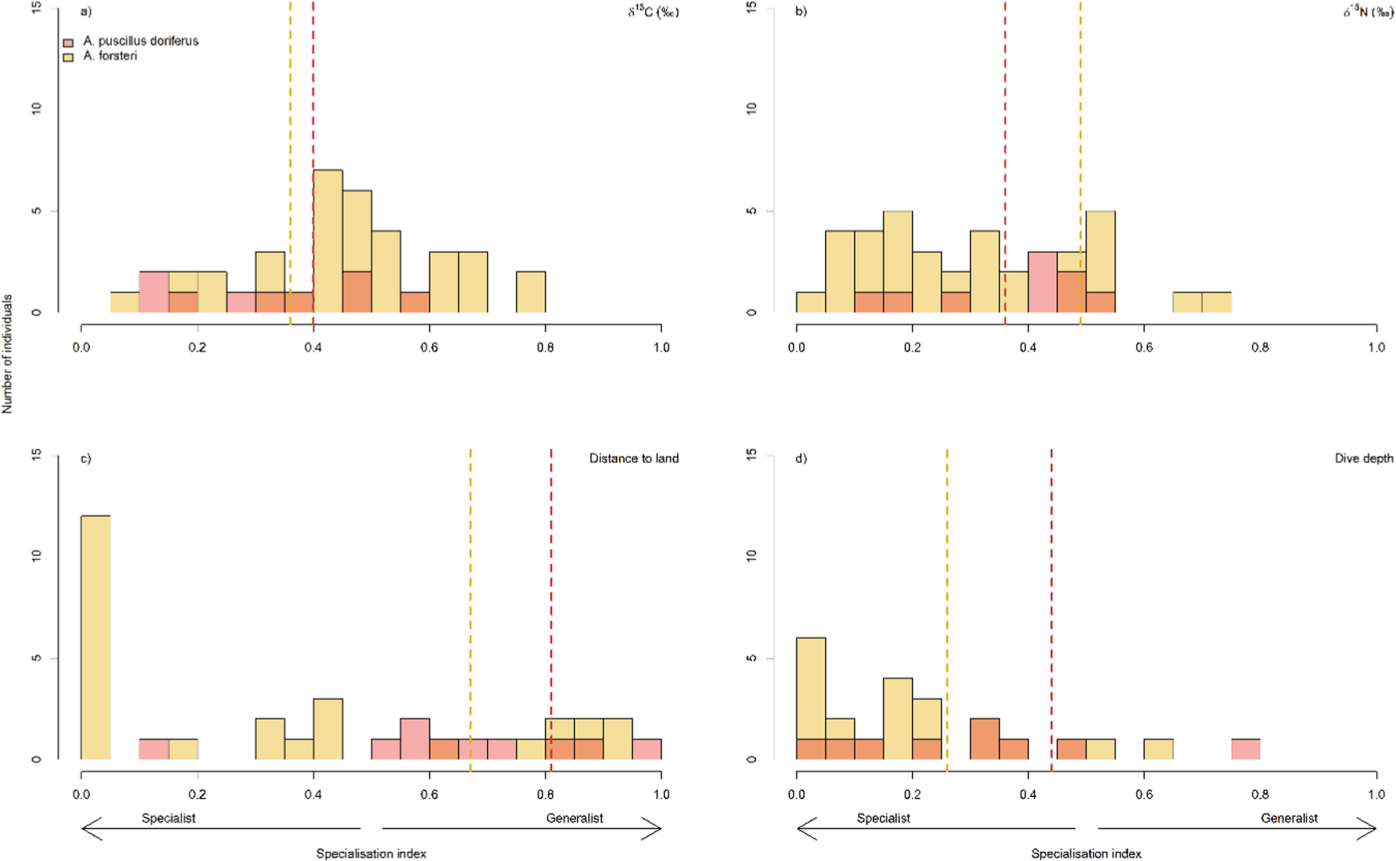
Density plot of specialisation index (SI) in δ^13^C and δ^15^N values and spatial parameters for each individual male Australian fur seals (*Arctocephalus pusillus doriferus*, AuFS, *red*) and New Zealand fur seals (*A. forsteri*, NZFS, *yellow*). Sample size for isotopic data *N* = 9 AuFS and *N* = 35 NZFS and for spatial data *N* = 10 AuFS and *N* = 35 and 39 for dive and location NZFS data, respectively. *Vertical dotted lines* show the population-level SI (from Table 1)

The individual specialisation index (SI) of δ^13^C values, δ^15^N values and dive depth for AuFS and NZFS indicated these male fur seals were specialists in each of these niche dimensions (*P* < 0.001; Table 2). However, there was high variability in the SI amongst individuals for each species (Fig. 3), with some individuals tending towards the generalist end of the spectrum but most individuals at the specialist end of the spectrum. For distance to land, AuFS were generalists and NZFS were specialists, though both species had high variability in the SI amongst individuals with their values spread across the SI spectrum (Fig. 3). There were a relatively large number of highly specialised male NZFS for ‘distance to land’; 12 individuals with SI values <0.05. These individuals include some who travelled off the continental shelf into deep water during each week, and other individuals who only moved between islands and the coastline (i.e. remained very close to land).

**Table 2:** Population-level isotope niche space statistics (δ^13^C and δ^15^N) and spatial niche space statistics (distance to land and dive depth) from Bayesian Standard Ellipse Area (SEA_b_) analysis and kernel density utilisation distributions (UD) of male Australian fur seals (*A. pusillus doriferus*) and New Zealand fur seals (*A. forsteri*)

Population-level statistics	*A. pusillus doriferus*	*A. forsteri*
*Isotope niche space*	***(N* = *9)***	***(N* = *35)***
SEA_b_ 40% area (‰^2^)^a,d^	1.0 ± 0.11	1.0 ± 0.04
SEA_b_ 95% area (‰^2^)^a,e^	5.8	6.1
SEA_b_ 40% overlap (%)^a,d^	5.8 ± 1.04	5.7 ± 4.12
SEA_b_ 95% width δ^13^C (‰)^b^	−16.2 to −14.7	−16.3 to −15.2
SEA_b_ 95% width δ^15^N (‰)^b^	15.6 to 17.4	14.1 to 16.2
*Spatial niche space*	***(N* = *10)***	***(N* = *35)***
SEA_b_ 40% area (‰^2^)^a, d^	1.4 ± 0.2	3.5 ± 0.2
SEA_b_ 95% area (‰^2^)^a, e^	8.2	20.9
SEA_b_ 40% overlap (%)^a, d^	48.3 ± 10.0	19.6 ± 4.4
SEA_b_ 95% width distance to land (km)^b^	2.3 to 23.5	1.0 to 53.1
SEA_b_ 95% width dive depth (m)^b^	17.4 to 164.4	9.2 to 67.3
*Spatial niche space, horizontal only*	***(N* = *10)***	***(N* = *39)***
Area of 95% UD (km^2^)	17,478	72,375
Overlap of 95% UD (%)	71	17
Area of 50% UD (km^2^)	1577	1109
Overlap of 50% UD (%)	52	73

Isotopic space calculated from 3 mm segments from one vibrissae per individual. Spatial niche calculated from weekly mean statistics per individual. The overlap statistic indicates the percentage of a species’ ellipse area that is shared with the other species

^a^Mean ± SE and range are calculated at the individual level (i.e. mean of each individual’s average value for its whisker segments or weekly movement data)

^b^Range of values

^c^Spatial niche parameters were log-transformed, and subsequently their back-transformed estimates of means are accompanied by 95% confidence intervals

^d,e^A sample of 50 SEA_b_ were used to calculate 40% areas and overlap, and one sample of 1 SEA_b_ was used to calculate 95% SEA_b_ areas and SEA_b_ widths

There were no correlations between an individual’s SI in any dimension and its body length (Online Resource; Table S2); all *P* > 0.05. An individual’s SI in one dimension (e.g. δ^13^C) was not related to its SI in another dimension (e.g. δ^15^N).

## Discussion

Our results indicate that male Australian and New Zealand fur seals that are reoccupying the north-eastern extent of their respective ranges share a broad ecological niche space but have significant partitioning in isotopic and spatial dimensions of their niche, despite expectations of ecological release from high intraspecies competition. Given their broad niches, it was not surprising that males of both species showed high levels of individual specialisation in isotopic and spatial niche space, particularly given their increased intraspecies competition over recent decades. Highly specialised individuals in isotopic space were not necessarily highly specialised in spatial niche space, further emphasising their diverse strategies for niche partitioning. There was support for a link between foraging mode and individual specialisation, as for other fur seals, though unexpectedly high specialisation for epipelagic NZFS males suggests exceptions may be apparent amongst marginal populations of a species’ distribution.

### Niche partitioning

As populations increase in size so can intraspecies competition for favourable food resources (i.e. rate of energy gain), which should drive individuals to broaden their niche (diet and/or foraging behaviour) to maintain optimal foraging (MacArthur and Pianka 1966; Roughgarden 1972; Bolnick 2001; Svanbäck and Bolnick 2007). Amongst marine predators, increased intraspecies competition has been associated with broader dietary niche and foraging niche attributed to the need to access different prey, prey at deeper depths and greater distances from their colony (Lewis et al. 2001; Kuhn et al. 2014; Ratcliffe et al. 2018). Along the same lines, subantarctic fur seals in a large population that has reached carrying capacity had a wider niche than those from a smaller population that is still increasing (Kernaléguen et al. 2015a). In contrast, at their range margin where population sizes are still small, these male fur seals continued to display a broad dietary niche (δ^15^N values) and spatial niche (horizontal and vertical behaviour), and this is consistent with an earlier dietary analysis of fur seal scats (Hardy et al. 2017). Whilst sample size was small, particularly for Australian fur seals, SIBER is robust to both this and unbalanced samples between populations (Jackson et al. 2011). Ideally, *n* > 20 would shrink confidence intervals as per Syvaranta et al. (2013), but whilst our sample size is small, the use of serially growing tissue for each individual would mitigate this by increasing the accuracy of each individuals’ niche values. The results presented here are also consistent with earlier dietary analyses and the size and consistency of the difference in niche space between species supports this analysis of niche space. Perhaps more discrete spatial niches of both individuals and the species as a whole might emerge if we could limit analyses to foraging areas and exclude travel behaviour. Individuals may expand their foraging niche in response to high intra- or high interspecific competition or decreased availability of food resources (Chiaradia et al. 2003; Moleón et al. 2009; Prati et al. 2021) and these factors typify a species’ range margin (MacArthur 1984; Case et al. 2005; Guo et al. 2005). Therefore, individuals may need to maintain a broad niche when moving between their range core and margins to mitigate different types of competition (intra and interspecies) and variable abundance of favourable prey throughout their distribution.

Interspecific competition was expected at this range margin, with two congeneric species living in sympatry. Elsewhere, large, established populations of two other otariids, South American fur seals and southern sea lions, show significant partitioning in their isotope niche space (Riverón et al. 2021). However, at the range margins populations are small so interspecific competition should be low thereby allowing these species to share the most favourable resources (in terms of energy gain) and overlap niche space. These male fur seals did indeed overlap in the prey source of primary productivity (δ^13^C values), trophic level of their prey (δ^15^N values; Kelly 2000; Davenport and Bax 2002) and spatial niche space, consistent with males of both species being high order predators that frequently return to land to rest and digest, and have foraging habitat at a range of depths (Page et al. 2005a; Hardy et al. 2017; Knox et al. 2017; Salton et al. 2021). Although the two species had overlapping niches, they had clear partitioning in their dietary niche and dive behaviour, with AuFS typically feeding on higher trophic level prey or on prey with different δ^15^N at the base of the food web in their feeding location than NZFS (based on δ^15^N values; Davenport and Bax 2002) and generally diving deeper than NZFS. Similar means of niche partitioning (different dietary composition and foraging behaviour) were found between sympatric female AuFS and NZFS at a breeding colony (Hoskins et al. 2017) and between sympatric male AuFS and NZFS at a New Zealand fur seal breeding colony (Page et al. 2005a). Unlike small populations at range margins, at breeding colonies this partitioning is expected because the larger populations suggest that absolute competition (intra and interspecific competition combine) should be higher (Shaughnessy et al. 2015; McIntosh et al. 2018). It is possible that competition in the core of their range drove niche partitioning ancestrally, and neither species is plastic enough in foraging to relax feeding constraints when seasonally present at the range margin, even in the absence of resource limitations. This idea could be explored more completely if similar individuals from populations at the range centre and range margin were sampled concurrently and then both intra and interpopulation comparisons could be considered in addition to intra and interspecies comparisons.

### Individual specialisation

Niche expansion can occur when all individuals of a population exploit a wider niche or via increased between-individual variation in niche dimensions. The latter is termed the Niche Variation Hypothesis (Van Valen 1965) and has supporting quantitative evidence from numerous taxa (Bolnick et al. 2007). Consistent with this hypothesis, fur seal populations that feed only on a few prey species are often made up of generalist individuals and populations with a broad dietary niche often have high levels of individual specialisation (Kernaléguen et al. 2015a; Riverón et al. 2021), including Australian fur seals (Kernaléguen et al. 2015b; this study) and New Zealand fur seals (this study). In addition to the Niche Variation Hypothesis, the level of individual specialisation in a population can be positively related to population density (Svanbäck and Persson 2004; Svanbäck and Bolnick 2005, 2007; Tinker et al. 2008), presumably because smaller populations have less intraspecies competition driving niche expansion, which appears to be the case for some fur seals (Franco-Trecu 2014; Kernaléguen et al. 2015a). Therefore, individuals at range margins, within small populations, may have lower individual specialisation than conspecifics at the range core. In contrast to this, the level of individual specialisation in δ^13^C values and δ^15^N values amongst male AuFS at this range margin (0.40 and 0.36, respectively) was higher (more specialised) compared to male AuFS in the core of the species’ range (0.93 and 0.56, respectively; Kernaléguen et al. 2015b). Some of this disparity could be associated with the shorter temporal scale used to measure individual specialisation in our study (10 whisker segments, rather than whole vibrissae), which often exaggerates the apparent level of individual specialisation (Araújo et al. 2007; Novak and Tinker 2015; Kernaléguen et al. 2016), though niche size and overlap were similar for the 10 segment and whole whisker datasets (Online Resource; Fig. S1). Alternatively, this disparity may provide support for the hypothesis that behavioural differences exist between dispersers and residents, with dispersers having higher heterogeneity in behaviour that supports population expansion into novel environments (Cote et al. 2010).

The level of specialisation in niche dimension varied amongst individuals, suggesting disproportionate effects of the drivers of specialisation on individuals. Accordingly, we tested whether the level of individual specialisation in one niche dimension was linearly related to specialisation in other niche dimensions, and found this was not the case for any of the four niche dimensions. Therefore, a seal may show a highly specialised dietary niche (δ^15^N values) but forage across a range of habitats to access their prey (less specialised spatial niche). Alternatively, a seal may have principally foraged epipelagically in inshore habitat (specialised spatial niche) on a broad range of prey (less specialised dietary niche). This suggests that individuals respond to the drivers of specialisation in different ways, potentially specialising in various niche dimensions but not necessarily all of them. This emphasises the behavioural plasticity of individuals to selection pressures and highlights the importance of considering multiple niche dimensions when assessing ecological drivers and consequences of individual specialisation.

Whilst species-specific foraging modes were apparent (i.e. benthic verses pelagic), both species were specialists in isotopic and spatial niche space based on Monte Carlo resampling tests for a null, generalist population. Benthic environments typically have a high diversity of prey, with each prey species having relatively low abundance, compared to the low diversity of pelagic species that are highly abundant (Gray 1997). Therefore, the benthic environment offers greater opportunity and motivation (e.g. to alleviate competition for limited resources) for predators to specialise on particular prey, whereas the pelagic environment has less potential and perhaps motivation for individuals to diverge from the average population diet. Empirical evidence shows pelagic foraging fur seals using offshore habitats have narrow isotopic niches, with generalist individuals and low specialisation, whilst benthic foraging fur seals using inshore habitats have a broader population isotopic niche with many specialist individuals (Riverón et al. 2021). Male AuFS on the range margin are consistent with this prediction from other fur seals, displaying benthic inshore foraging and consisting of mostly individual niche specialists. However, male NZFS movement behaviour was typical of epipelagic foraging, and they also had high individual specialisation. These male NZFS exploited predominantly inshore but also offshore habitats, and some male NZFS remained close to the coast displaying an apparent benthic foraging mode. Ecological diversification often occurs in marine mammals that forage in inshore areas (Wolf et al. 2008; Chilvers and Wilkinson 2009; Aurioles-Gamboa et al. 2013), perhaps due to the greater diversity of isotopic pathways in coastal environments (Ray 1991) and greater habitat complexity (Sequeira et al. 2018). Given these populations are small, perhaps there is some interspecies competition release that creates space for some male NZFS to exploit the benthic and inshore habitats, thereby increasing potential for inter-individual diversification. This may change as populations increase, and male AuFS come to dominate the inshore environment and NZFS forage more epipelagically further from the coast (Page et al. 2006).

### Ecological implications

As species expand their range into new habitat, they must compete for resources with the native community, which already compete amongst themselves. The size of a community can influence the level of niche overlap, with increasing number of species associated with less overlap (Pianka 1974), and if the community is sufficiently large it can prevent newly introduced species from becoming established (Case 1990). This has implications for the success of biological invasions (MacArthur 1984; Freed and Cann 2014), and potentially the recovery and range expansion associated with conservation efforts of a native species. Given the smaller populations of both species at this expanding range margin, there was potential for high niche overlap associated with competition release. Somewhat contradictory, the niche overlap and individual specialisation between and within these male fur seals suggests there is available niche for each of these species and potential for further mitigation of inter and intraspecies competition, and therefore, potential for population growth and range expansion. Indeed, prior to this study both populations of fur seals in Australia had positive population trajectories (Shaughnessy et al. 2015; McIntosh et al. 2018). Ongoing assessments of niche partitioning and individual specialisation within and between these sympatric and congeneric species at this range margin will further develop ecological understanding of the mechanisms for successful population growth and range expansion and should consider the role of a rapidly warming environment. These assessments would benefit from concurrent sampling of individuals within the core parts of their species range to better quantify the mechanisms operating throughout different parts of a species range.

Individual specialisation and behavioural plasticity provide opportunities for a population to adapt to environmental change (Brent 1978; Bolnick et al. 2003; Tuomainen and Candolin 2011; Edelaar and Bolnick 2019). Accordingly, the high individual specialisation amongst these male fur seals may contribute to their successful re-occupation of this margin of their range amidst extreme rates of ocean warming (Ridgway 2007) and a dense human population. However, species have physiological limits, for example, otariids in temperate regions are sensitive to high temperatures (Gentry 1973; Ladds et al. 2017), and thermal energetic costs are often higher for pups and juveniles (Liwanag 2010). Species are also limited by habitat needs, in this case particular terrestrial features at haul-out and breeding sites (Ryan et al. 1997; Stevens and Boness 2003), and several of their haul-out sites at this margin of their range are currently not zoned as protected areas (Salton et al. 2021). Therefore, whilst males have reoccupied this part of the species’ range, these additional limitations could influence the successful reestablishment of a breeding population and future occupation by males.

Furthermore, ocean warming is altering prey distribution and abundance and thereby the habitat uses of marine predators (Schumann et al. 2013; Amador‐Capitanachi et al. 2020; Evans et al. 2020; Niella et al. 2020, 2021; d’Entremont et al. 2021; Florko et al. 2021). There have been recent losses of habitat and habitat-forming species at this margin of the seals’ range (Wernberg et al. 2011). Thus, whilst these predators demonstrate capability to exploit a dynamic environment and a high level of adaptiveness to change, a rapidly warming environment presents several risks that could limit population growth and expansion at this margin of their range. These risks would compromise the success of current conservation efforts that have seen these species reoccupy parts of their historic range. To mitigate such compromises, we encourage actions that support species to adapt to climate change (Hobday et al. 2016; Roberts et al. 2017; Miller et al. 2018; Wilson et al. 2020). In particular, both a larger and targeted network of protected areas on land and at sea (Salton et al. 2021), and informed, dynamic management of both these predators and their changing prey base (Nelms et al 2021).

## Supplementary Information

Below is the link to the electronic supplementary material.

Supplementary file1 (DOCX 1023 KB)

## Data Availability

The datasets generated during and/or analysed during the current study are available from the corresponding author on reasonable request.
