# Correction: Niche partitioning and individual specialisation in resources and space use of sympatric fur seals at their range margin

**DOI:** 10.1007/s00442-024-05622-y

**Published:** 2024-09-18

**Authors:** Marcus Salton, Vincent Raoult, Ian David Jonsen, Matt Carr, Robert Harcourt

**Affiliations:** 1 School of Natural Sciences, Macquarie University, North Ryde, NSW 2109 Australia; 2 Australian Antarctic Division, Department of Climate Change, Energy, the Environment and Water, Kingston, TAS 7050 Australia; 3 School of Environmental and Life Sciences, University of Newcastle, Ourimbah, 2258 Australia; 4 Department of Primary Industries, Jervis Bay Marine Park, NSW 2540 Australia; 5 Present Address: Biodiversity Conservation Trust, Coffs Harbour, NSW 2450 Australia

**Correction: Oecologia (2024) 204:815–832** 10.1007/s00442-024-05537-8

In this article Matt Carr at affiliation 'Department of Primary Industries, Jervis Bay Marine Park, New South Wales 2540, Australia' was missing from the author list. Correct version of author list as follow.

Marcus Salton · Vincent Raoult · Ian David Jonsen · Matt Carr · Robert Harcourt

Matt's affiliations are as follows:

Department of Primary Industries, Jervis Bay Marine Park, New South Wales 2540, Australia

Present address: Biodiversity Conservation Trust, Coffs Harbour, New South Wales 2450, Australia

## Data Availability

The custodians of the raw data used in this study are Macquarie University (Montague Island study site; available through Australian Ocean Data Network https://portal.aodn.org.au) and Marine Parks, New South Wales Department of Primary Industries (Jervis Bay study site). Derived data supporting the findings of this study are available from the corresponding author MS on request. Montague Island seal data can be sourced from Australia's Integrated Marine Observing System (IMOS) - IMOS is enabled by the National Collaborative Research Infrastructure Strategy (NCRIS).

