# Supplementary material for: Niche partitioning and individual specialisation in resources and space use of sympatric fur seals at their range margin
Source: Oecologia. 2024 Apr 3;204(4):815–32. doi: 10.1007/s00442-024-05537-8 (PMC11062968; doi:10.1007/s00442-024-05537-8)
Supplement: Supplementary file 1 — Supplementary file1 (DOCX 1023 KB) [file 442_2024_5537_MOESM1_ESM.docx]

**ELECTRONIC SUPPLEMENTARY MATERIAL**

**Titl**e: Niche partitioning and individual specialisation in resources and space use of sympatric fur seals at their range margin

**Author Names:** Marcus Salton^1,3^, Vincent Raoult^1,2^, Ian Jonsen^1^, Robert Harcourt^1^

**Affiliations:**

^1^School of Natural Sciences, Macquarie University, North Ryde, New South Wales 2109, Australia

^2^School of Environmental and Life Sciences, University of Newcastle, Ourimbah 2258, Australia

^3^Present address: Australian Antarctic Division, Department of Climate Change, Energy, the Environment and Water, Kingston, Tasmania 7050, Australia

**Corresponding author email**: [marcussalton@gmail.com](mailto:marcussalton@gmail.com)

**Journal:** Oecologia

**Figure Legends**

**Online resource Figure S1**. Isotopic biplots for each of the four niche parameters for each individual male Australian fur seals (*A. pusillus doriferus*, red; N=10) and New Zealand fur seals (*A. forsteri*, yellow; N=35) calculated with three different datasets (one per row). Dataset 1: a) and b) average values per individual. Dataset 2: c) and d) first 10 whisker segments or first 10 weeks of movement data, which is used in the analysis and presented in Figure 1. Dataset 3: e) and f) all whisker segements and all weeks of movement data. Standard Ellipse Area (40%) showing first 50 draws for clarity.

**Online resource Figure S2.** Different circadian patterns in dive frequency and dive depth between male Australian fur seals (*A. pusillus doriferus*, red; N=10) and New Zealand fur seals (*A. forsteri*, yellow; N=35). a) and c) dive frequency per hour of day (Australian Eastern Standard Time; AEST). b) and d) average dive depth per hour of day (mean per individual). e) and f) modelled values of dive frequency and dive depth, respectively, per diel period (mean individual, per week). Modelled significant differences relatively to reference factor level (diel period ‘day’) are represented by *** (P<0.001) or ns (not significant).

**Online resource Figure 1**


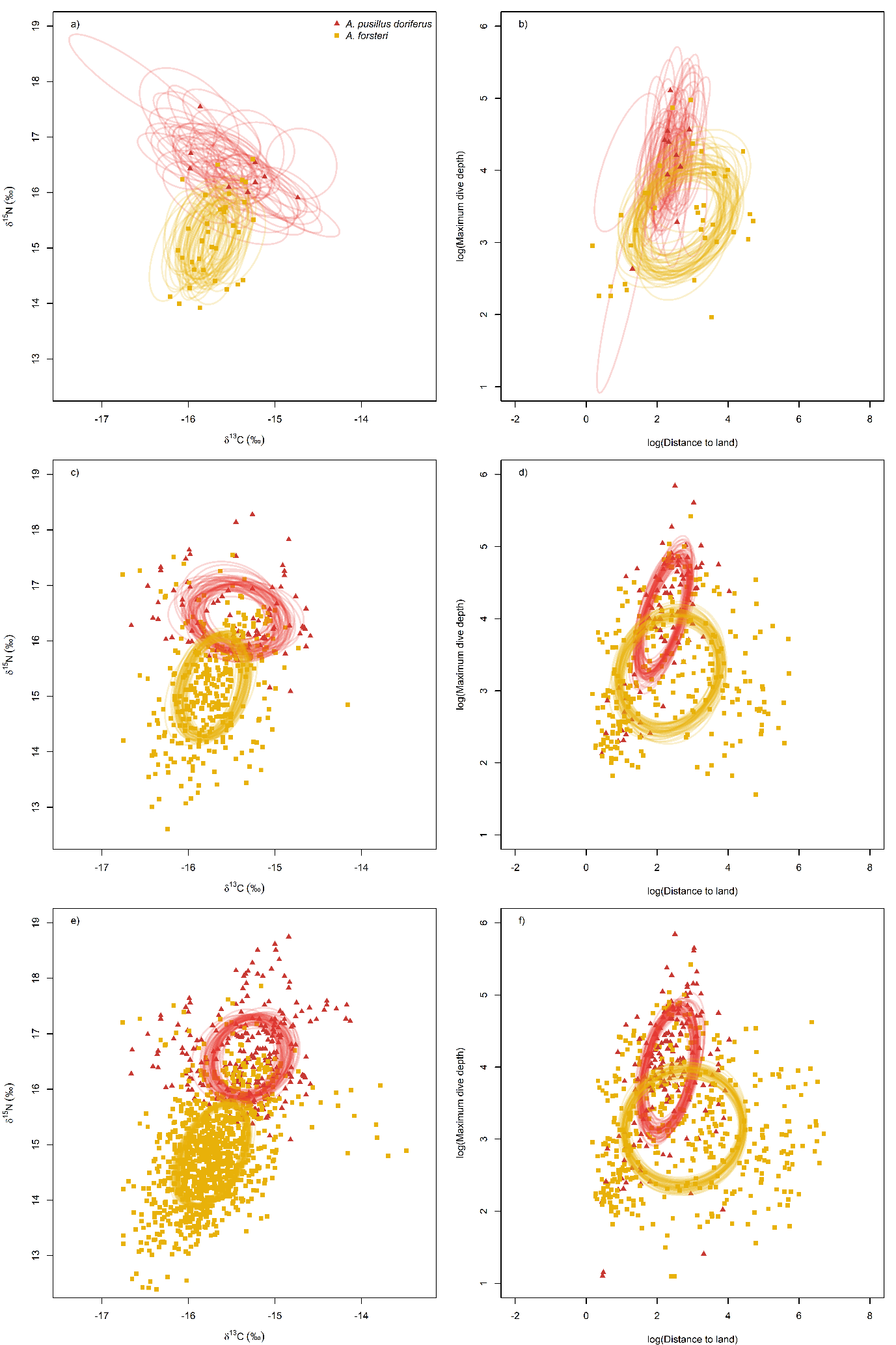


**Online resource Figure 2**


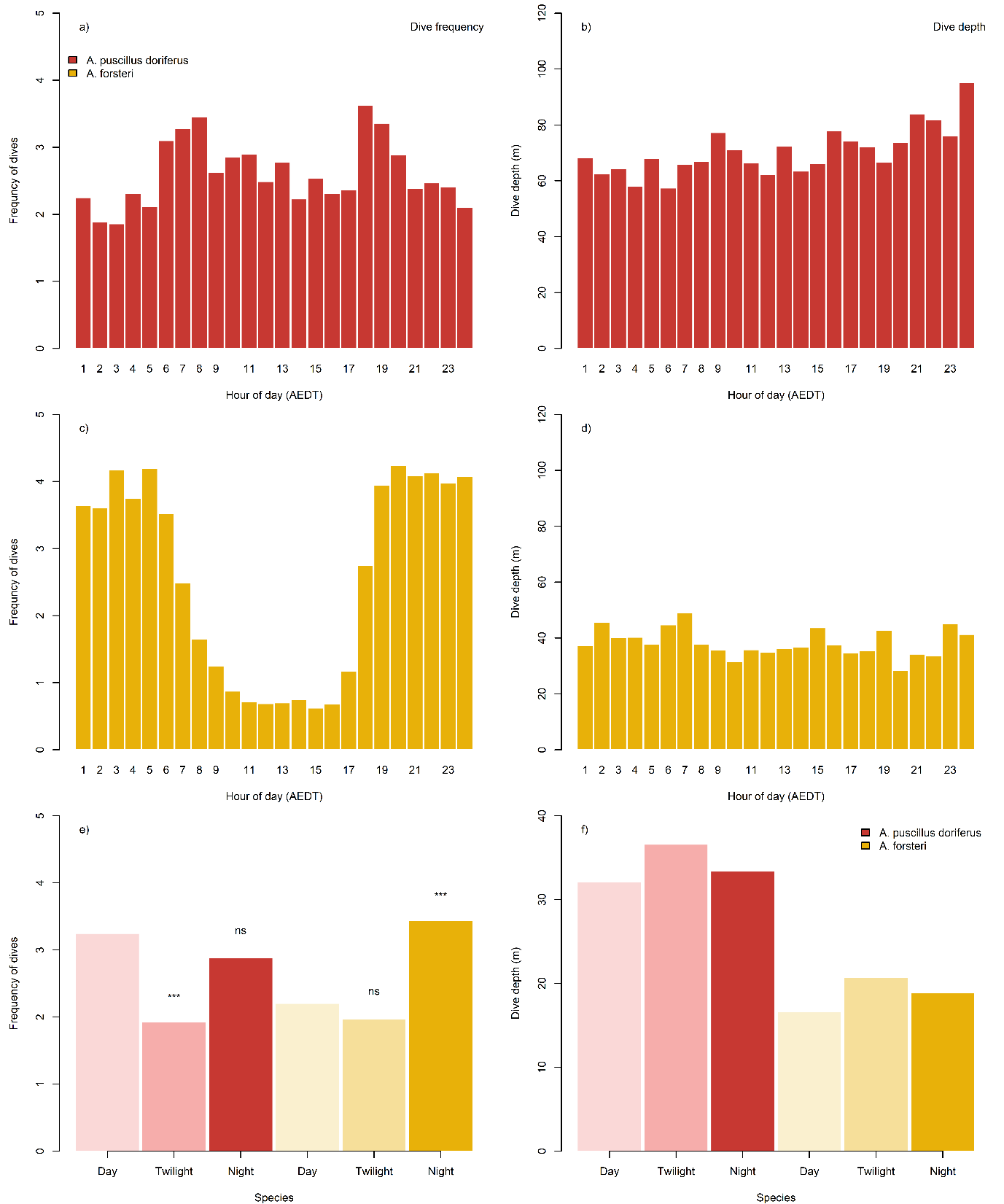


**ESM Table Legends**

**Online resource Table S1.** Model results for diel pattern in dive frequency and dive depth (using means per week for each individual) of male Australian fur seals (*A. pusillus doriferus*; N=10) and New Zealand fur seals (*A. forsteri*; N=35), including models tested (top) and modelled fixed effects (bottom). Dive depth was log transformed to normalise residuals (log values presented). Levels of fixed effects were tested against the reference level ‘Day’. Diel was not significant for dive depth, so the fixed effect levels were not tested.

**Online resource Table S2.** Linear model results comparing individual specialisation indices of male Australian fur seals (AuFS; N=10) and New Zealand fur seals (NZFS; N=35) in isotopic space (δ^13^C and δ^15^N) and spatial niche space (Dive depth and Distance to land), and compared to their body size (Length).

**Online resource Table S3.** Summary of male fur seals (Australian, aufs, N=10; New Zealand, nzfs, N=35) sampled (i.e. whisker) and/or tracked (with a Wildlife computer Mk10, Jervis Bay, ‘jb’; or SMRU, Montague Island, ‘mi’) between 2011 and 2014. Whiskers were cut into 3 mm segments: some whole whiskers others only the ten segments closest to the root of the whisker (most recent). Whiskers were sampled when a tracking device was attached, unless it was a dead seal (had died within days of collecting the whisker).

**Online resource Table 1**

|  |  | *A. pusillus doriferus* | |  | *A. forsteri* | |  |
| --- | --- | --- | --- | --- | --- | --- | --- |
| Models tested |  |  |  |  |  |  |  |
| Response | Fixed effects | logLik | AIC | P | logLik | AIC | P |
| Dive frequency | Diel | -1456.7 | 2931.4 |  | -4897.6 | 9813.2 |  |
|  | Null | -1471.8 | 2957.5 | < 0.001 | -4927.8 | 9869.6 | < 0.001 |
|  |  |  |  |  |  |  |  |
| Dive depth | Diel | -10065 | 20151 |  | -29528 | 59076 |  |
|  | Null | -10066 | 20149 | 0.399 | -29530 | 59076 | 0.111 |
| Modelled fixed effects | |  |  |  |  |  |  |
| Response | Diel period | Estimate (SE) | z | P | Estimate (SE) | z | P |
| Dive frequency | Day | 3.2 (0.2) | 21.4 |  | 2.2 (0.2) | 11.9 |  |
|  | Twilight | -1.3 (0.1) | -12.1 | <0.001 | -0.2 (0.1) | -1.7 | 0.082 |
|  | Night | -0.4 (0.3) | -1.3 | 0.202 | 1.2 (0.2) | 7.4 | <0.001 |
|  |  |  |  |  |  |  |  |
| Dive depth | Day | 3.5 (0.3) | na |  | 2.9 (0.2) | na |  |
|  | Twilight | 0.1 (0.2) | na |  | 0.2 (0.1) | na |  |
|  | Night | 0.04 (0.2) | na |  | 0.1 (0.1) | na |  |

**Online resource Table 2**

| Response | Predictor | Species | *T* value | *P* value |
| --- | --- | --- | --- | --- |
| *δ^15^N* | Length | AuFS | -2.694 | 0.054 |
| *δ^15^N* | Length | NZFS | -0.669 | 0.508 |
| *δ^13^C* | Length | AuFS | -1.074 | 0.343 |
| *δ^13^C* | Length | NZFS | 0.968 | 0.341 |
| Dive depth | Length | AuFS | -1.948 | 0.123 |
| Dive depth | Length | NZFS | 1.747 | 0.091 |
| Distance to land | Length | AuFS | 0.906 | 0.416 |
| Distance to land | Length | NZFS | -1.962 | 0.059 |
| *δ^13^C* | *δ^15^N* | AuFS | 1.217 | 0.291 |
| *δ^13^C* | *δ^15^N* | NZFS | 1.745 | 0.091 |
| Dive depth | *δ^15^N* | AuFS | 1.366 | 0.244 |
| Dive depth | *δ^15^N* | NZFS | -0.723 | 0.475 |
| Distance to land | *δ^15^N* | AuFS | 0.027 | 0.980 |
| Distance to land | *δ^15^N* | NZFS | 0.539 | 0.594 |
| Dive depth | *δ^13^C* | AuFS | 0.337 | 0.753 |
| Dive depth | *δ^13^C* | NZFS | -1.321 | 0.196 |
| Distance to land | *δ^13^C* | AuFS | 0.681 | 0.533 |
| Distance to land | *δ^13^C* | NZFS | -1.408 | 0.169 |
| Distance to land | Dive depth | AuFS | -1.271 | 0.273 |
| Distance to land | Dive depth | NZFS | 0.045 | 0.964 |

**Online resource Table 3**

| ID | Location | Species | Year | Body length (cm) | Date deployment/  sample | Data types | Deployment duration (d) | N# dives |
| --- | --- | --- | --- | --- | --- | --- | --- | --- |
| JB-1-2011 | jb | nzfs | 2011 | 100 | 21-Jun-2011 | Loc | 201 | NA |
| JB-2-2011 | jb | nzfs | 2011 | 80 | 23-Jun-2011 | Loc | 30 | NA |
| JB-3-2011 | jb | nzfs | 2011 | 75 | 4-Jul-2011 | Loc | 91 | NA |
| JB-4-2011 | jb | nzfs | 2011 | 90 | 7-Jul-2011 | Loc | 60 | NA |
| JB-2-2012 | jb | nzfs | 2012 | 100 | 28-Jun-2012 | SI, Loc, Div | 166 | 4439 |
| JB-3-2012 | jb | nzfs | 2012 | 90 | 28-Jun-2012 | SI, Loc, Div | 113 | 2541 |
| JB-8-2013 | jb | nzfs | 2013 | 70 | 19-Jul-2013 | SI, Loc, Div | 166 | 3828 |
| JB-11-2013 | jb | nzfs | 2013 | 90 | 10-Jul-2013 | SI, Loc, Div | 187 | 3314 |
| JB-12-2013 | jb | nzfs | 2013 | 150 | 11-Jul-2013 | SI, Loc, Div | 154 | 1844 |
| JB-15-2013 | jb | nzfs | 2013 | 100 | 30-Jul-2013 | SI, Loc, Div | 10 | 40 |
| JB-16-2013 | jb | nzfs | 2013 | 100 | 9-Aug-2013 | SI, Loc, Div | 141 | 3810 |
| JB-17-2013 | jb | nzfs | 2013 | 110 | 22-Aug-2013 | SI, Loc, Div | 168 | 1458 |
| JB-10-2013 | jb | nzfs | 2013 | 90 | 9-Jul-2013 | SI, Loc, Div | 118 | 2328 |
| JB-7-2013 | jb | nzfs | 2013 | 100 | 19-Jun-2013 | SI, Loc, Div | 207 | 6229 |
| JB-14-2013 | jb | nzfs | 2013 | 100 | 26-Jul-2013 | SI, Loc, Div | 106 | 2017 |
| JB-9-2013 | jb | aufs | 2013 | 250 | 20-Jun-2013 | SI, Loc, Div | 156 | 3662 |
| JB-13-2013 | jb | aufs | 2013 | 200 | 25-Jul-2013 | SI, Loc, Div | 190 | 3836 |
| MI-3-2012 | mi | nzfs | 2012 | 162 | 23-Jun-2012 | SI, Loc, Div | 259 | 674 |
| MI-4-2012 | mi | nzfs | 2012 | 183 | 23-Jun-2012 | SI, Loc, Div | 233 | 887 |
| MI-1-2012 | mi | nzfs | 2012 | 154 | 12-Jun-2012 | SI, Loc, Div | 178 | 665 |
| MI-2-2012 | mi | nzfs | 2012 | 152 | 12-Jun-2012 | Loc, Div | 146 | 1071 |
| MI-5-2012 | mi | nzfs | 2012 | 165 | 23-Jun-2012 | Loc, Div | 69 | 340 |
| MI-7-2012 | mi | nzfs | 2012 | 185 | 23-Jun-2012 | SI, Loc, Div | 67 | 226 |
| MI-60-2012 | mi | nzfs | 2012 | NA | 21-Jul-2012 | SI | NA | NA |
| MI-16-2013 | mi | nzfs | 2013 | 167 | 28-May-2013 | SI, Loc, Div | 120 | 558 |
| MI-4-2013 | mi | nzfs | 2013 | 165 | 25-May-2013 | SI, Loc, Div | 115 | 370 |
| MI-3-2013 | mi | nzfs | 2013 | 163 | 25-May-2013 | SI, Loc, Div | 94 | 367 |
| MI-1-2013 | mi | nzfs | 2013 | 162 | 25-May-2013 | SI, Loc, Div | 50 | 112 |
| MI-5-2013 | mi | nzfs | 2013 | 160 | 25-May-2013 | SI, Loc, Div | 49 | 234 |
| MI-9-2013 | mi | nzfs | 2013 | 155 | 26-May-2013 | SI, Loc, Div | 29 | 234 |
| MI-7-2013 | mi | nzfs | 2013 | 167 | 26-May-2013 | SI, Loc, Div | 25 | 164 |
| MI-12-2013 | mi | nzfs | 2013 | 170 | 27-May-2013 | SI, Loc, Div | 17 | 122 |
| MI-8-2013 | mi | nzfs | 2013 | NA | 26-May-2013 | SI | NA | NA |
| MI-2-2014 | mi | nzfs | 2014 | 164 | 2-Aug-2014 | SI, Loc, Div | 35 | 18 |
| MI-1-2014 | mi | nzfs | 2014 | 174 | 2-Aug-2014 | SI, Loc, Div | 15 | 70 |
| MI-5-2014 | mi | nzfs | 2014 | 138 | 3-Aug-2014 | SI, Loc, Div | 91 | 191 |
| MI-4-2014 | mi | nzfs | 2014 | 176 | 3-Aug-2014 | SI, Loc, Div | 70 | 137 |
| MI-3-2014 | mi | nzfs | 2014 | 155 | 3-Aug-2014 | SI, Loc, Div | 23 | 38 |
| MI-8-2014 | mi | nzfs | 2014 | 157 | 4-Aug-2014 | SI, Loc, Div | 78 | 183 |
| MI-7-2014 | mi | nzfs | 2014 | 155 | 4-Aug-2014 | SI, Loc, Div | 58 | 193 |
| MI-6-2014 | mi | nzfs | 2014 | 172 | 4-Aug-2014 | SI, Loc, Div | 30 | 50 |
| MI-9-2014 | mi | nzfs | 2014 | 150 | 5-Aug-2014 | SI, Loc, Div | 61 | 184 |
| MI-10-2014 | mi | nzfs | 2014 | 162 | 5-Aug-2014 | SI, Loc, Div | 34 | 104 |
| MI-6-2012 | mi | aufs | 2012 | 180 | 24-Jun-2012 | SI, Loc, Div | 141 | 876 |
| MI-9-2012 | mi | aufs | 2012 | 177 | 25-Jun-2012 | Loc, Div | 109 | 480 |
| MI-8-2012 | mi | aufs | 2012 | 190 | 24-Jun-2012 | Loc, Div | 62 | 253 |
| MI-6-2013 | mi | aufs | 2013 | 173 | 25-May-2013 | SI, Loc, Div | 185 | 1223 |
| MI-10-2013 | mi | aufs | 2013 | 180 | 26-May-2013 | SI, Loc, Div | 166 | 1114 |
| MI-14-2013 | mi | aufs | 2013 | 200 | 28-May-2013 | Loc, Div | 152 | 338 |
| MI-15-2013 | mi | aufs | 2013 | 180 | 28-May-2013 | SI, Loc, Div | 110 | 638 |
| MI-13-2013 | mi | aufs | 2013 | NA | 27-May-2013 | Loc, Div | 47 | 352 |
| MI-11-2013 | mi | aufs | 2013 | NA | 27-May-2013 | SI | NA | NA |
| MI-2-2013 | mi | aufs | 2013 | NA | 25-May-2013 | SI | NA | NA |
| MI-60-2013 | mi | aufs | 2013 | NA | 22-Aug-2013 | SI | NA | NA |
